# Facing danger: how do people behave in times of need? The case of adult attachment styles

**DOI:** 10.3389/fpsyg.2014.01452

**Published:** 2014-12-10

**Authors:** Tsachi Ein-Dor

**Affiliations:** Interdisciplinary Center Herzliya, School of PsychologyHerzliya, Israel

**Keywords:** attachment, anxiety, avoidance, defensive behavior, social defense theory

## Abstract

Bowlby’s (1982) attachment theory has generated an enormous body of research and conceptual elaborations. Although attachment theory and research propose that attachment security provides a person with many adaptive advantages, during all phases of the life cycle, numerous studies indicate that almost half of the human species can be classified as insecurely attached or insecure with respect to attachment. It seems odd that evolution left humans in this vulnerable position, unless there are some advantages to individuals or groups, under at least some conditions, of anxious and avoidant attachment styles. I argue that a social group containing members with different attachment patterns may be more conducive to survival than a homogeneous group of securely attached individuals because each attachment disposition has specific adaptive advantages that promote the survival of the individual and people around him or her when facing threats and perils. In making this argument, I extend the scope of attachment theory and research by considering a broader range of adaptive functions of insecure attachment strategies, and present data to support my argument.

## BACKGROUND

As illuminated in cave painting, humans have faced threats and danger throughout the eons (Valladas et al., 2001). To survive, animals commonly employ fight-or-flight responses in times of need (Cannon, 1929; Jansen et al., 1995). Humans, however, are wanting in their physiological ability to effectively fight threats, and fall short in their ability to flight by climbing or running afoot. For example, humans are almost bare of body hair (Bergman, 2004) and its protective attributes against cuts, bruises, and bites (e.g., Blanchard, 2009), which make them vulnerable when fighting threats. Humans were probably perfected by evolution to solve the worriment of survival by cooperating with others and utilizing the strength of numbers (Axelrod and Hamilton, 1981; Cosmides, 1989; Brewer and Caporael, 1990; Axelrod, 2006). To date, however, research on human defensive reactions to threats has focused mainly on individual-level responses such as people’s attentional bias toward signals of threats (e.g., Brandtstädter et al., 2004), people’s responses to threat scenarios (Perkins and Corr, 2006) and people’s actions and reactions in life-endangering events (see Mawson, 2012 for a comprehensive review). In the present paper, I present social defense theory (SDT; Ein-Dor et al., 2010), in an attempt to bridge the gap in the literature on human defensive behavior by suggesting one possible group-level process by which people promote the likelihood of surviving perilous events.

Social defense theory suggests that we ought to acknowledge the effects of other people around us on our responses to threats and on our ability to prevail dicey challenges. Specifically, SDT proposes that some people are more perceptive of threat-related cues and tend to detect threats quicker and more accurately than others. Other people are compulsively self-reliant and upon detection of threats tend to employ self-protective actions more rapidly and effectively than others. Still other people are better at massing collective efforts and leading group actions because they are more relationship-oriented than others. Because each of these responses promotes survival in a unique way, I contend that groups comprising these three styles of people (i.e., more heterogeneous groups with respect to people’s personality and related action tendencies) will be more effective when dealing with threats than more homogeneous groups because they combine the abilities for early detection of threats, rapid responses, and effective cooperation. According to SDT (Ein-Dor et al., 2010), these three personality dispositions are the manifestation of people’s attachment orientations (see Mikulincer and Shaver, 2007 for an extensive review).

## ATTACHMENT THEORY

According to attachment theory (Bowlby, 1973, 1980, 1982), humans possess a mammalian innate psycho-biological system – the attachment system – that was perfected by evolution to promote the survival of infants, although it is remains active “from the cradle to the grave” (Bowlby, 1982, p. 208). It motivates the individual to seek proximity to significant others (attachment figures) when he or she feels a need for protection and care. When attachment figures respond sympathetically to a person’s needs over a long series of interactions, they fosters a sense of attachment security, which in time formulates into a trait-like disposition of security about the self, others and the world. In adulthood, secure people respond to threats either by relying on internal resources to regulate stress and to maintain high self-esteem and self-efficacy or by seeking concrete support from others or collaborating with them to regain safety and to restore a sense of security (Shaver and Mikulincer, 2002).

Conversely, when attachment figures often fend off bids for support or respond unreliably to a person’s needs, they unintentionally foment one out of two chronic states of insecurity – avoidance, marked by abysmal independence, lack of trust in others, and maintaining a defensive pretense of security while employing cognitive and emotional avoidance especially in times of need; and anxiety, marked by symbiotic dependence and immutable sense of strain while maintaining constant hypervigilance to threats and intensified negative affectivity (see Mikulincer and Shaver, 2007, for a review). Social and personality psychologists generally conceptualize adult attachment dispositions as regions in a continuous two-dimensional space and not as typologies (e.g., Brennan et al., 1998). One dimension relates to avoidance and the second to attachment-related anxiety. Attachment security is defined by low scores on both anxiety and avoidance.

The dominant perspective regarding attachment security is that secure people enjoy adaptive advantages in all fabric of life compared with people high on anxiety and/or avoidance (Mikulincer and Shaver, 2007). For example, secure people endorse fewer psychopathologies (Ein-Dor and Doron, in press), adopt more constructing coping strategies with relationship-related conflicts and stress (Mikulincer et al., 2002a), and tend to be viewed by potential partners as more attractive (e.g., Klohnen and Luo, 2003). These advantages of security and related benefits lead researchers to ponder why approximately half of the human population of earth are insecure with respect to attachment. Belsky et al. (2010) and Del Giudice and Belsky (2011) were the first to suggest that attachment anxiety and avoidance have adaptive benefits such as earlier menarche in females (also see Chisholm et al., 2005) that allows earlier reproduction in stressful and risky environments in which waiting for optimal conditions might result in failing to reproduce.

Research has indicated, however, that the probable selection pressure that caused the emergence of the attachment behavioral system in mammalian evolution was survival-related and not early reproduction (Bowlby, 1973, 1982; Mikulincer and Shaver, 2007; Ein-Dor et al., 2010; Ein-Dor, 2013). Various physical and psychological threats such as loud noises, darkness, fatigue, and illness activate the attachment system (Bowlby, 1973), and the behavioral and cognitive outcomes of the system such is proximity seeking increase the likelihood of protection and survival (Mikulincer et al., 2000, 2002b). In keeping with these findings, Ein-Dor et al. (2010) and his colleagues proposed SDT, which suggests that each of the three major attachment dispositions – security, anxiety, and avoidance – awards unique adaptive advantages for the individual and for people around her or him that increase the likelihood of surviving perilous events.

## SOCIAL DEFENSE THEORY

A decade ago, Nettle’s (2006, p. 625) has contended that personality variations can be understood in terms of tradeoffs among fitness costs and benefits: “Behavioral alternatives can be considered as tradeoffs, with a particular trait producing not unalloyed advantage but a mixture of costs and benefits such that the optimal value for fitness may depend on very specific local circumstances”. SDT (Ein-Dor et al., 2010) adopts Nettle’s perspective and proposes that security and insecurity dispositions alike endow adaptive advantages that increase the likelihood of survival while also incurring distinct disadvantages that might hinder survival unless they are complemented by contributions from people with different attachment dispositions.

### ADVANTAGES AND DISADVANTAGES OF SECURE INDIVIDUALS’ DEFENSIVE REACTIONS

Attachment research has indicated that secure people tend to lead team efforts and promote the success of their social group by collaborating with others in times of need. For example, secure individuals endorse greater prosocial and task-oriented leadership motivations and lower self-enhancing and self-reliance motivations than their more insecure counterparts (Davidovitz et al., 2007; Hinojosa et al., 2014). Secure leaders are also apprised by their followers as demonstrating higher efficacy in emotion-focused situations and task-focused ones (Davidovitz et al., 2007). As teammates they work more effectively with other group members when solving problems and facing challenges (e.g., Rom and Mikulincer, 2003; Molero et al., 2013). These advantages are believed to be the manifestation of a sense of security that was developed in past supportive experiences with attachment figures (Mikulincer and Shaver, 2007) and which comprises core beliefs regarding the safeness of the world and people in it. These optimistic, solacing mental representations promote self-palliative reappraisals of threats, which help secure people to outperform insecure individuals in many daily and challenging situations alike (Mikulincer and Shaver, 2007).

A sense of security is not always linked with actual physical security, however. In times of danger, it can be maladaptive if it clogs the recognition of threats and slows down rapid, effective responses. For example, Mawson (2012, p. 233) has indicated that “while mass panic (and/or violence) and self-preservation are often assumed to be the natural response to physical danger and perceived entrapment… the typical response to a variety of threats and disasters is not to flee but to seek the proximity of familiar persons and places”. Therefore, secure individuals tend to seek proximity to others (Waters and Waters, 2006; Mikulincer et al., 2009), even if this is not always the safest strategy. Adopting a schema of security about the self, others, and the world may, therefore, incur two prominent disadvantages: (a) delayed perception of danger and (b) slower employment of effective defensive behaviors in response to threats and danger.

Sime (1983, 1985), in keeping with Mawson’s (1978) suggestions, examined these disadvantages in a police-reports-based study of reactions to a fire in a large coastal resort on the Isle of Man, Great Britain, 1973. Sime showed that people who reported being close to family members were less likely to react to early signs of danger such as noises and shouts. Rather, they tended to react only after witnessing unambiguous cues of danger, such as people running while holding fire extinguishers, smoke, and flames – which usually means a loss of precious time in successfully escaping the situation. Other studies of survivors’ behavior during perilous events have also indicated that people who reported being close to family members perceived that they were in danger slower than people who were alone in the situation (Aguirre et al., 1998; Köster et al., 2011). According to SDT, the tendency to mainly react to clear signs of threats and not to take heed to earlier cues of danger characterizes secure people (Ein-Dor et al., 2010).

Regarding slower employment of effective defensive behaviors in times of need, research has indicated that security with respect to attachment may be linked with non-optimal reactions in times of danger. For example, Mawson (2012, p. 153) noted that in combat situations, “what may be important for the individual soldier is maintaining proximity with his fellows, even though this may involve moving into situations of greater physical danger”. Studies on natural disasters have also indicated that “people tended to turn to and protect loved ones rather than flee from the threat” (Form and Nosow, 1958, p. 26) and that “traditional family ties often keep individual members in the danger zone until it is too late” (Hill and Hansen, 1962, p. 217).

In keeping with SDT, research has indicated that the advantages of secure people come into play in their better leadership qualities and their ability to coordinate group activities. Nevertheless, these advantages are partially countervail by their sluggish perceptiveness of actual threats and their somewhat imperfect reactions to danger because of their will to stay close to people around them. This suggests that secure people’s inclination to focus on ongoing tasks and chores irrespective of mounting danger may impede their survival. Attentiveness to early signs of danger and hasty fight-or-flight responses may be necessary to evade disaster. SDT suggests that being high on either attachment avoidance or anxiety might confer such adaptive abilities and counterpoise the disadvantages of attachment security when facing perilous events.

### ADVANTAGES AND DISADVANTAGES OF PEOPLE HIGH ON ATTACHMENT ANXIETY

People high on attachment anxiety often appraise their own functioning in groups as imperfect and are judged by others as falling short in their ability to effectively lead team efforts in completing various tasks (Davidovitz et al., 2007). They take work less seriously than their secure counterparts and make fewer contributions to a team and of poorer quality (Rom and Mikulincer, 2003). Despite these shortcomings, the hypervigilant strategies that anxious people adopt when dealing with threats might nevertheless promote their survival and benefit others in their social surrounding: They sensitively monitor the environment for threats and upon detection of danger they seek support by actively calling on others for help and by overacting their emotions (Cassidy and Kobak, 1988; Feeney and Noller, 1990). Ein-Dor et al. (2010) named these behavioral tendencies sentinel behavior.

According to SDT, the sentinel behavior is stemming from a self-schema that guides anxious people’s responses in times of need. It comprises default action tendencies that cause people high on attachment anxiety “(a) to remain vigilant with respect to possible threats, especially in unfamiliar or ambiguous situations; (b) to react quickly and strongly to early, perhaps unclear cues of danger (e.g., unusual noises, shuﬄing feet, shouts); (c) to alert others about the imminent danger; (d) if others are not immediately supportive, to heighten efforts to get them to provide support; and (e) to minimize distance from others when coping with a threat” (Ein-Dor et al., 2011a, p. 2).

The benefits of sentinel behavior is apparent in many species of animals. For example, African elephants (Soltis et al., 2014) and chimpanzees (e.g., Schel et al., 2013), among other species of animals such as birds (Evans et al., 1993) and fish (Smith, 1992), produce shrill alarm calls when they detect a potential threat as predators. Humans may also benefit from the hyperactivating strategies of people high on anxiety in similar ways.

The first evidence in favor of this notion linked attachment anxiety with heightened accessibility to core components of the sentinel schema – noticing danger quicker than others and warning them about the danger (Ein-Dor et al., 2011a). For example, when participants were asked to write a story about a TAT-like (Thematic Apperception Test; Murray, 1943) card with a scary scenario, those higher on attachment anxiety composed stories with more sentinel-related narratives. After reading a story about a person who behave in a sentinel way, participants who scored higher on attachment anxiety were more likely to generate more inferences about this person and his personality.

Attachment anxiety was later linked with actual sentinel-related behavior in times of need (Ein-Dor et al., 2011b). Specifically, the behavior of small groups of three people were observed in an experimentally manipulated threatening situation: a room progressively filling up with non-toxic smoke from what seems like a malfunctioning computer. In line with SDT, groups higher on attachment anxiety detected the presence of smoke quicker than less anxious groups. Specifically, 1-point increase in anxiety was linked with 11.5 s decrease in detection time. In addition, the person with the highest score on anxiety detected the presence of smoke in the room more often than predicted by chance alone (Ein-Dor et al., 2011a). In a complementary self-report-based research, participants were asked to report on the first action that they are likely to take on various threat scenarios (Ein-Dor and Perry-Paldi, 2014). Results indicated that attachment anxiety qualified the effects of situational features (e.g., degree of dangerousness and clarity of the threat) to increase the likelihood of sentinel (e.g., yelling) and fear-related behaviors (e.g., running away).

Aside from establishing a link between attachment anxiety and reaction to potential life-engendering threats, people high on attachment anxiety were also found to have a tendency to deliver a warning message without delay (Ein-Dor and Orgad, 2012). Using a designated software, participants were led to believe that they accidently activated a Trojan horse that completely erased the experimenter’s hard drive and possibly the campus’s server. Participants were then asked to alert the computer technicians about the hazard. On their way, the researchers created four behavioral settings in which they tried to delay the participants from delivering the warning message (e.g., a confederate who asked them to help her completing a short questionnaire). In line with SDT, results indicated that high attachment anxiety was linked with fewer delays.

Research has also shown that attachment anxiety is associated with the ability to accurately detect social-based threats. For example, people high on attachment anxiety are better apt in foretelling their partners’ true thoughts and feelings in situations that pose a threat to the relationship such as when partners rate an attractive opposite-sex person (Simpson et al., 1999, 2011). People high on attachment anxiety were also better at detecting cues of interpersonal deceit (Ein-Dor and Perry, 2014). Specifically, participants watched a series of seven video clips in which people retold the events they experienced the day before. In some of the clips the speaker was honest and in some – dishonest. Participants were asked to appraise whether the person in the clip lied or told the truth. In an additional study, semi-professional poker players completed a self-report questionnaire measuring attachment dispositions and then they participated in a poker tournament that was held outside campus. Results indicated that people higher on attachment anxiety were more accurate in detecting deceitful statements, and that players higher on anxiety won greater amount of money during a game of poker, which relates to an ability to call opponents’ bluffs. Taken together, research has supported SDT’s premise regarding the possibility that people high on attachment anxiety adopt sentinel-related cognitions and behaviors that promote survival.

### ADVANTAGES AND DISADVANTAGES OF PEOPLE HIGH ON ATTACHMENT-RELATED AVOIDANCE

People high on attachment avoidance relegate appraisals of threats and downgrade sensations of pain and vulnerability (e.g., Fraley and Shaver, 1997). Therefore, they are usually less vigilant to signs of danger and tend to recognize the extent of threat later than others (Ein-Dor et al., 2010). They tend to appraise team cohesion as more fractured than others and are often appraised by others as less apt to lead because of withered emotional abilities (Davidovitz et al., 2007). They do not tend to collaborate with others and, hence, they do not perform well as teammates (Rom and Mikulincer, 2003). In times of need, they are compulsively self-reliant (Bowlby, 1973) and tend to take self-protective actions that promote their own interests (Feeney and Collins, 2001), a reaction tendency that Ein-Dor et al. (2010) named rapid fight-or-flight behavior. As a result, while anxious and secure individuals focus their attention on the whereabouts of significant others around them, without focusing quickly enough on how to evade the progressive threat, avoidant people are able to discover a way to effectively deal with the threat.

The asocial tendencies of people high on avoidance might actually help people around them eluding danger. Suppose that an avoidant person is in a shopping mall engrossed by flames. To save her or himself, he or she will take quick protective actions to espy the best route to escape or to quickly extinguish the fire. These behaviors increase the avoidant person’s survival chances but might also save other people’s lives. For example, the sight of people running from danger can motivate the escape of others around them and unintentionally save lives (e.g., Mawson, 2012). As Marshall (1947, pp. 145–146) noted regarding the military behavior during World War II: “It can be laid down as a general rule that nothing is more likely to collapse a line of infantry than the sight of a few of its number in full and unexplained flight to the rear... One or two or more men made a sudden run to the rear which others in the vicinity did not understand... In every case the testimony of all witnesses clearly [indicated] that those who started the run... had a legitimate or at least a reasonable excuse for the action”. Aside from promoting the motivation for escape, people who flee before others do must clear an escape route of possible obstacles and, thus, others can enjoy an open route to follow. Taken together, people high on avoidance may increase their own and their group members’ chances of survival in times of need.

According to SDT, the asocial behavior of avoidant individuals stems from a rapid fight-or-flight schema that comprises the following action tendencies: “(a) minimize the importance of threatening stimuli; (b) when danger is clearly imminent, take quick self-protective action, either by escaping the situation or by taking action against the danger; and (c) at such times, do not worry about coordinating one’s efforts with those of other people” (Ein-Dor et al., 2011a, p. 3).

The first evidence in favor of this notion linked attachment-related avoidance with the following core narratives of the rapid fight-or-flight schema when writing a story about a scary scenario: (a) escaping a perilous event without helping others, (b) acting without collaborating or deliberating with others, and (c) reacting quickly. After reading a story about a person who behave in a rapid fight-or-flight way, participants high on attachment avoidance generated more inferences about the person’s behaviors and thoughts than people low on avoidance.

Attachment avoidance was later linked with actual rapid fight-or-flight behavior in times of need (Ein-Dor et al., 2011b). Specifically, research has indicated that the typical response to a room progressively filling up with smoke was fleeing to the adjunct corridor. In line with SDT, groups higher on attachment avoidance was quicker to escape the room than more secure groups, and were appraised by judges as more effective in dealing with the situation. In a complementary self-report-based research, attachment avoidance was found to qualify the effects of situational features (e.g., degree of dangerousness and clarity of the threat) to increase the likelihood of rapid-responder (e.g., attacking; which relates to fight responses), fear-related (e.g., running away; which relates to flight reactions), and anxiety-related (e.g., risk assessment) reactions (Ein-Dor and Perry-Paldi, 2014). Taken together, research has supported SDT’s notion that attachment-related avoidance is associated with rapid fight-or-flight cognitions and behaviors.

### GROUP COMPOSITION AND ITS ASSOCIATION WITH EFFECTIVENESS WHEN DEALING WITH THREAT

In the course of evolution, humans lived in relatively small groups or tribes of kin and often faced threats and perils. As individuals, we are lacking in our ability to survive: we have a fragile body, which hinders our ability to effectively fight threats, and we were evolved to walk on two legs, which limits our ability to effectively escape threats. We survived by utilizing the strength of numbers and by facing perils as a group. SDT contends that to survive we needed several abilities that one person can never hope to have: heightened vigilance to threats and danger, quick responses to threats once they are detected, and calm and calculated collective efforts to overcome the threats. An effective response to threats could only be achieved by the combining efforts of people with different attachment dispositions. According to SDT, each of the three major styles of attachment dispositions – security, anxiety, and avoidance – have both unique adaptive advantages that promote survival and disadvantages that might hinder survival. Heterogeneous groups with respect to attachment dispositions should be more sensitive to early signs of threat by utilizing the sentinel abilities of anxious members; act quickly in response to threats without much deliberation by utilizing the rapid fight-or-flight abilities of avoidant members; and manage complex group-level tasks by utilizing the leadership and social-oriented abilities of secure members. Accordingly, a group comprising all three styles of attachment patterns may benefit from the combined abilities of each disposition and offset their shortcomings. Therefore, such groups might be superior to other groups in dealing with threats and survival problems.

In support of this proposition, heterogeneous groups with respect to attachment patterns were appraised by external judges as dealing more effectively with a room gradually filling up with smoke than more homogenous groups (Ein-Dor et al., 2011b). Heterogeneity in attachment patterns was also found to promote the success of work teams. Specifically, teams’ heterogeneity in attachment anxiety and avoidance scores was related to better academic grades (Lavy et al., 2014). This latter finding was moderated by teams’ cohesion, however. Heterogeneity was linked with better performance only among teams that were able to construct high sense of cohesion. In other words, heterogeneity could be a double-ended sword.

Individuals with either anxiety or avoidance dispositions could present a social challenge to groups’ dynamics: People high on attachment anxiety because of their hyperactivation tendencies are clingy, needy, vexed, and fearful and are constantly seeking approval of others, sometimes by being intrusive (Smith et al., 1999). People high on attachment avoidance might neglect the needs of others and keep their distance of others, which may hinder effective communication within the group (Smith et al., 1999; Rom and Mikulincer, 2003). These tendencies may cause conflicts between team members and reduce teams’ socio-emotional functioning (Pelled et al., 1999; although teams’ objective performance might still be high). Nevertheless, when insecure team members are in a reassuring environment that accept them and let them feel safe and trusted, their challenging relationship-related perceptions and behaviors might be turned into advantages.

## CONCLUDING COMMENTS

Social defense theory was devised to suggest one possible group-level process by which people promote the likelihood of surviving perilous events. It is based on the proposition that different attachment dispositions bring different abilities to a group – sentinel, rapid fight-or-flight, and leadership abilities – rendering the group superior to other groups in dealing with threats and survival problems. Pending on receiving additional empirical support, SDT may have important implications for theory and research concerning human defensive behaviors, group dynamics, threat detection, and adaptive benefits of personality diversity.

## Conflict of Interest Statement

The authors declare that the research was conducted in the absence of any commercial or financial relationships that could be construed as a potential conflict of interest.
